# Exploratory analysis of prognostic factors and hematologic dynamics in unresectable ESCC treated with concurrent versus interval immune checkpoint inhibitors combined with (chemo)radiotherapy: a multicenter real-world study

**DOI:** 10.3389/fimmu.2026.1728912

**Published:** 2026-03-12

**Authors:** Xiaohan Zhao, Hesong Wang, Wang Jie, Bingliang Li, Yaowen Zhang, Chenyu Wang, Yatian Liu, Pudong Qian, Jianzhong Cao, Qing Hou, Yuanji Xu, Zhongmei Lin, Xianghua Ye, Yaqi Song, Jiahua Lv, Shuchai Zhu, Wenbin Shen

**Affiliations:** 1 Department of Radiation Oncology, Hebei Medical University Fourth Hospital, Shijiazhuang, China; 2 Hebei Normal University, College of Life and Science, Shijiazhuang, China; 3 Hebei Medical University, School of Basic Medical Sciences, Shijiazhuang, China; 4 Department of Radiation Oncology, Anyang Tumor Hospital, The Affiliated Anyang Tumor Hospital of Henan University of Science and Technology, Anyang, China; 5 Department of Radiation Oncology, Nanjing Medical University Affiliated Cancer Hospital & Jiangsu Cancer Hospital & Jiangsu Institute of Cancer Research, Nanjing, China; 6 Department of Radiation Oncology, Shanxi Province Cancer Hospital, Cancer Hospital Affiliated to Shanxi Medical University, Taiyuan, China; 7 Department of Radiation Oncology, Clinical Oncology School of Fujian Medical University, Fujian Cancer Hospital, Fuzhou, China; 8 Department of Radiation Oncology, The First Affiliated Hospital, Zhejiang University School of Medicine, Hangzhou, China; 9 Department of Oncology, Affiliated Hospital of North Sichuan Medical College, Nanchong, China; 10 Department of Radiotherapy, Sichuan Clinical Research Center for Cancer, Sichuan Hospital Cancer & Institute, Sichuan Cancer Center, University of Electronic Science and Technology of China, Chengdu, China

**Keywords:** concurrent, esophageal squamous cell carcinoma (ESCC), immune checkpoint inhibitors (ICI), interval, radiotherapy

## Abstract

**Background:**

The optimal integration of radiotherapy (RT) and immune checkpoint inhibitors (ICI) for esophageal squamous cell carcinoma (ESCC) remains undefined. This study aimed to evaluate treatment patterns, hematologic dynamics, and prognostic factors in patients receiving combined RT and ICI.

**Methods:**

We conducted a multicenter retrospective analysis of 426 patients with unresectable ESCC treated with RT and ICI, with and without chemotherapy. Survival outcomes were compared between concurrent and Interval (chemo)radiotherapy ((C)RT) and ICI strategies. Hematologic parameters, including lymphocyte counts and the systemic inflammation score (SIS), were dynamically assessed at baseline, during RT, and post-RT. Prognostic factors for overall survival (OS) and progression-free survival (PFS) were analyzed using univariate and multivariate Cox regression models.

**Results:**

Concurrent and Interval (C)RT-ICI strategies demonstrated comparable OS and PFS. Concurrent (C)RT-ICI was associated with a more pronounced decline in lymphocyte counts and a slightly higher incidence of adverse events, though generally manageable. Patients with isolated lymph node metastases achieved survival similar to those without metastasis and significantly better than those with organ metastases. During concurrent(C)RT-ICI, lymphocyte counts showed the most significant decline but gradually recovered 1–2 months after radiotherapy. Notably, SIS measured 1–2 months after RT emerged as a superior independent prognostic indicator for OS.

**Conclusions:**

In real-world practice, concurrent (C)RT-ICI is a safe and feasible treatment option for ESCC, though associated with greater lymphocyte suppression. During ICI treatment, SIS assessed after RT may serve as a simple and reliable prognostic biomarker. Patients with isolated nodal metastases appear to derive substantial benefit from (C)RT-ICI, warranting further validation in prospective randomized trials.

## 1 Background

Esophageal cancer remains one of the major malignancies threatening global health. According to GLOBOCAN 2022, it ranks seventh in incidence and sixth in mortality among all cancers worldwide (1). Esophageal squamous cell carcinoma (ESCC) is the predominant histological subtype, accounting for more than 85% of all esophageal cancer cases globally (2). For unresectable esophageal cancer, chemoradiotherapy(CRT) is the standard treatment modality; however, even in patients with localized disease, therapeutic outcomes remain unsatisfactory (3).

The advent of immune checkpoint inhibitors (ICI) has provided new opportunities in the management of esophageal cancer. Multiple phase III randomized controlled trials have demonstrated significant survival benefits when ICI are combined with chemotherapy as first-line therapy for advanced disease (4, 5). In unresectable locally advanced or oligometastatic cases, radiotherapy(RT) plays a critical role. Recent retrospective studies have suggested encouraging outcomes when RT is combined with ICI (6, 7). Nevertheless, most of these studies were limited by small sample sizes and incomplete clinical information. Key issues that concern clinicians—such as the optimal timing and dosage of RT in relation to ICI, as well as the dynamics of hematologic parameters—remain inadequately addressed, limiting the ability to derive prognostic insights regarding combined RT and ICI.

Furthermore, many clinicians remain skeptical about the efficacy and safety of Concurrent RT and ICI modality. While in the concurrent setting, radiotherapy may reduce cytotoxic T lymphocyte counts, which may lead to increased therapeutic toxicity (8, 9). A strategy that avoids simultaneous administration, defined as Interval RT and ICI, might appear safer; however, it may prolong the treatment course, delay disease control, negatively impact patient well-being, and reduce treatment adherence.

To address these uncertainties, this study collected clinical data from 9 medical centers across China on patients with ESCC treated with first-line RT-ICI, combined with or without chemotherapy. The study focused on exploring treatment modalities (Concurrent vs. Interval), prognosis factors, associated toxicities, and dynamic changes in hematologic parameters, with the aim of providing clinically relevant insights to guide practice.

## 2 Methods

### 2.1 Study design and eligibility criteria

This multicenter exploratory study aimed to investigate prognostic factors in patients with ESCC treated with ICI in combination with RT. The study was approved by the Ethics Committee of the Fourth Hospital of Hebei Medical University and conducted in accordance with the principles of the Declaration of Helsinki. Data from consecutive patients who received ICI were collected from 9 cancer treatment centers across China. We collected data from patients treated between March 2018 and March 2023. Owing to the retrospective design, the requirement for informed consent was waived by the ethics committees of Hebei Medical University. The study was prospectively registered at ClinicalTrials.gov (NCT06478355).

Inclusion criteria

1. Pathologically confirmed ESCC, age ≥18 years;
2. Unresectable disease or patient refusal of surgery;
3. Receipt of first-line RT combined with ICI, both administered within the same line of treatment (either Interval or concurrently), with no prior antitumor therapy;
4. Interval between initiation of RT and ICI ≤6 months;
5. Completion of at least 80% of the prescribed RT dose and ≥2 cycles of ICI;
6. No severe hepatic, renal dysfunction, or bone marrow suppression;
7. Absence of contraindicating comorbidities for ICI.

Exclusion criteria

RT or ICI administered solely as salvage treatment after disease progression;

Missing data on treatment initiation or survival follow-up.

### 2.2 Treatment and grouping

Given the retrospective nature of the study, treatment regimens, including the choice of ICI, were not restricted. Patients who received ≥6 cycles of ICI were classified as having received ICI maintenance. To investigate the impact of treatment integration, patients were stratified into two groups based on whether ICI was administered during the RT course, regardless of whether chemotherapy was combined: the Concurrent (chemo)radiotherapy plus ICI group (Con (C)RT-ICI) and the Interval (chemo)radiotherapy plus ICI group (Int (C)RT-ICI).

### 2.3 Evaluation and follow-up

Overall survival (OS) was defined as the interval from the start of the first anticancer therapy (RT or ICI) to death from any cause or the date of the last follow-up. Progression-free survival (PFS) was calculated from the initiation of treatment to the first confirmed disease progression, death from any cause, or the last available follow-up, whichever occurred earlier. All participants received regular follow-up evaluations, including laboratory testing, chest and abdominal computed tomography (CT), esophagography, and ultrasound examinations of superficial lymph nodes. Positron emission tomography/computed tomography (PET/CT) or histopathological biopsy was performed when clinically indicated. During ICI, assessments were scheduled every 2–3 treatment cycles, and after completion of ICI, patients were reviewed every 1–3 months. Treatment-related adverse events occurring during or after therapy were graded using the National Cancer Institute’s Common Terminology Criteria for Adverse Events (CTCAE), version 5.0.

### 2.4 Collection of hematologic parameters and calculation of inflammation-nutrition indices

Baseline hematologic parameters were obtained prior to initiation of systemic treatment. Parameters during RT were collected within 2–4 weeks after the start of RT, and post-RT parameters were collected within 1–2 months after treatment completion. To minimize confounding by acute chemotherapy-related effects, blood samples were not collected within one week after chemotherapy administration. If multiple eligible measurements were available within the same time window, the result with the lowest lymphocyte count was selected for analysis, given its sensitivity to RT.

Inflammation and nutrition indices were calculated as follows:

LMR: Lymphocyte-to-Monocyte Ratio.

Systemic Inflammation Score (SIS): 0 (albumin ≥40 g/L and LMR ≥4.44), 1 (albumin <40 g/L or LMR <4.44), or 2 (albumin <40 g/L and LMR <4.44).

Prognostic Nutritional Index (PNI): albumin (g/L) + 5 × lymphocyte count (×10^9^/L).

Other indices such as NLR, PLR, and SII were not included, as all use lymphocyte counts as the denominator, and RT-induced lymphocyte fluctuations could introduce excessive statistical noise and compromise reliability.

### 2.5 Statistical analysis

Categorical variables were compared using the χ² test, or Fisher’s exact test when the expected frequency was <5. Continuous variables were expressed as mean ± standard deviation (SD) for normally distributed data or median (interquartile range) for non-normally distributed data. Differences between groups were assessed using the independent-samples t-test. Homogeneity of variance was tested with Levene’s test: if P > 0.05, the standard t-test was used; if P ≤ 0.05, Welch’s correction was applied. Paired-samples t-tests were used to compare hematologic parameters before, during, and after RT within the same patients, with normality of differences verified using the Shapiro-Wilk test.

Survival outcomes were analyzed using the Kaplan-Meier method, and differences between groups were assessed with the log-rank test. Median follow-up was estimated using the reverse Kaplan-Meier method. To mitigate immortal time bias, a 4-month landmark analysis was employed for the Kaplan-Meier assessment of ICI maintenance. Multivariable Cox proportional hazards regression was performed to identify independent prognostic factors for overall survival (OS) and progression-free survival (PFS), with results presented as hazard ratios (HRs) and 95% confidence intervals (CIs).Propensity score matching (PSM) at a 1:1 ratio was performed to balance Con (C)RT-ICI and Int (C)RT-ICI with Caliper width = 0.2.

All statistical analyses were conducted using R software (version 4.2, R Foundation for Statistical Computing, Vienna, Austria) and SPSS version 25.0 (IBM Corp., Armonk, NY, USA). Two-sided tests were used throughout, with statistical significance defined as P < 0.05.

## 3 Results

### 3.1 Baseline characteristics

**Figure 1:**
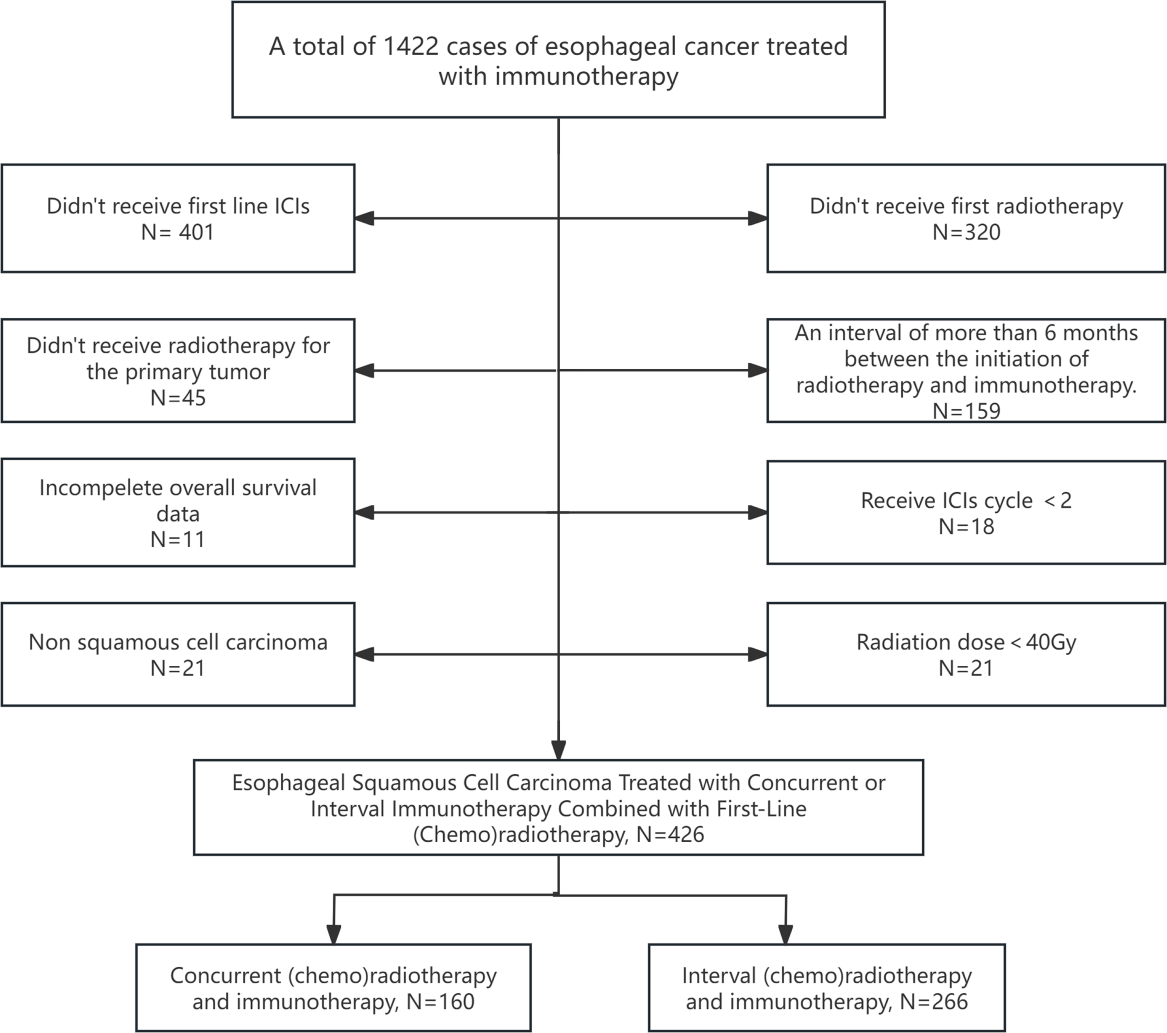
Flow chart.

A total of 1,422 patients were initially screened, and 426 patients were finally included in the study, comprising 160 in the concurrent CRT plus ICI group and 266 in the Interval group. The patient selection flowchart is shown in **Figure 1**. Among patients receiving first-line RT combined with ICI, 92% had either non-metastatic disease or lymph node-only metastasis, while only 8.2% presented with visceral metastases. Chemotherapy was administered to 96.5% of patients, with the majority (86.6%) receiving a platinum-based doublet regimen.

Compared with the concurrent group, the Interval group included a higher proportion of patients without metastases and more patients who received ICI maintenance. Patients in the concurrent group had better ECOG performance status, although the difference did not reach statistical significance. Detailed baseline characteristics are presented in **Table 1**.

**Table 1:** Baseline Characteristics.

Demographic factors	Total N=426	Con (C)RT-ICI N=160	Int (C)RT-ICI N=266	P
Gender				0.941
Male	323(75.8)	121(75.6)	202(75.9)	
Female	103(24.2)	39(24.4)	64(24.1)	
Age				0.515
<65	231(54.2)	90(56.3)	141(53.0)	
≥65	195(45.8)	70(43.7)	125(47.0)	
Smoking				0.459
Yes	234(57.2)	140(55.8)	94(59.5)	
No	175(42.8)	111(44.2)	64(40.5)	
Unknown	17			
ECOG score				0.052
0-1	383(89.9)	245(92.1)	138(86.3)	
≥2	43(10.1)	21(7.9)	22(13.8)	
Tumor Location				0.620
Cervical+Upper	142(33.3)	51(31.9)	91(34.2)	
Middle+Low	284(66.7)	109(68.1)	175(65.8)	
T stage				0.091
T1-2	61(15.3)	44(16.5)	17(10.6)	
T3-4	365(85.7)	222(83.5)	143(89.4)	
N stage				0.640
N0	52(12.2)	18(11.3)	34(12.8)	
N+	374(87.8)	142(88.7)	232(87.2)	
Metastasis status				0.041
No metastasis	222(52.1)	71(44.4)	151(56.8)	
Lymph node metastasis only	169(39.7)	75(46.9)	94(35.3)	
Organ metastasis	35(8.2)	14(8.8)	21(7.9)	
Chemotherapy modality				0.559
No chemotherapy	15 (3.5)	8(5)	7(2.6)	
Platinum-based doublet	369(86.6)	137(85.6)	232(87.2)	
Others doublet	4(0.9)	2(1.3)	2(0.8)	
Monotherapy	38(8.9)	13(8.1)	25(9.4)	
PD-L1 Status (CPS)				0.263
0	22(5.2)	5(3.1)	17(6.4)	
≥1	28(6.6)	9(5.6)	19(7.1)	
Unknown	376(88.3)	146(91.3)	230(86.5)	
ICI drug				0.133
Camrelizumab	139(32.6)	54(33.8)	85(32.0)	
Tislelizumab	59(13.8)	18(11.3)	41(15.4)	
Sintilimab	111(26.1)	35(21.9)	76(28.6)	
Pembrolizumab	43(10.1)	22(13.8)	21(7.9)	
Others	74(17.4)	31(19.4)	43(16.2)	
ICI maintenance				0.012
Yes	282(66.2)	94(58.8)	188(70.7)	
No	144(33.8)	66(41.2)	78 (29.3)	
Radiation Dose to Primary Esophageal Tumor				0.738
40–60 Gy	225(52.8)	86 (56.2)	139 (54.5)	
60 Gy	183(42.9)	67 (43.8)	116 (45.5)	

Con (C)RT-ICI: (Chemo)radiotherapy concurrent immune checkpoint inhibitors.

Int (C)RT-ICI: (Chemo)radiotherapy interval immune checkpoint inhibitors.

### 3.2 Exploratory analyze of RT and ICI sequence

Based on the chronological sequence of RT and ICI initiation, the Con (C)RT-ICI and Int (C)RT-ICI groups were further stratified into five distinct sub-patterns(**Table 2**). The specific composition of each subgroup and the median time intervals between the initiation of RT and ICI are detailed in the table below. Kaplan-Meier (KM) analysis demonstrated no significant differences in overall survival (OS) or progression-free survival (PFS) among these five subgroups (**Supplementary Figure 1**).

**Table 2:** Sequence of RT and ICI.

Group label	Treatment sub-patterns	N	Median interval (IQR), days	Clinical definition
Con (C)RT-ICI (n=160)	ICI first	96	57 (60)	ICI presence during RT
	Simultaneous Start RT + ICI (Defined as interval < 14 days)	53	6 (7)	ICI presence during RT
	RT first	41	47 (33)	ICI presence during RT
Int (C)RT-ICI (n=266)	ICI first	152	64 (52)	ICI-free during RT
	RT first	84	88 (62)	ICI-free during RT

Con (C)RT-ICI, (Chemo)radiotherapy concurrent immune checkpoint inhibitors.

Int (C)RT-ICI, (Chemo)radiotherapy interval immune checkpoint inhibitors.

### 3.3 Dynamic changes in hematologic parameters during radiotherapy and the impact of concurrent ICI

Hematologic parameters declined overall during RT, with the most pronounced decrease observed in lymphocyte counts. The baseline lymphocyte count was 1.41 ± 0.60 × 10^9^/L, which dropped to 0.57 ± 0.36 × 10^9^/L at mid-RT, representing a mean reduction of 0.84 × 10^9^/L (p < 0.001). Although lymphocyte counts recovered to 1.09 ± 0.96 × 10^9^/L within 1–2 months after RT, they remained below baseline, with a mean decrease of 0.33 × 10^9^/L (p < 0.001).

White blood cells decreased from 5.92 ± 2.29 × 10^9^/L at baseline to 4.50 ± 1.88 × 10^9^/L at mid-RT (p < 0.001) and partially recovered to 5.05 ± 2.58 × 10^9^/L post-RT, though still lower than baseline (p < 0.001). Neutrophil counts declined from 3.44 ± 1.60 × 10^9^/L to 2.85 ± 1.42 × 10^9^/L during treatment and increased to 3.21 ± 2.31 × 10^9^/L afterwards, with no significant differences compared to baseline (all p > 0.05). Monocytes decreased from 0.51 ± 0.20 × 10^9^/L to 0.39 ± 0.18 × 10^9^/L, then rose slightly to 0.43 ± 0.26 × 10^9^/L, both significantly lower than baseline (p < 0.01). Platelet counts declined from 215.6 ± 71.5 × 10^9^/L at baseline to 180.3 ± 64.4 × 10^9^/L mid-RT and slightly increased to 185.2 ± 92.8 × 10^9^/L post-RT but remained significantly below baseline (p < 0.001). In contrast, albumin and LDH showed no significant fluctuations across baseline, mid-, and post-RT (albumin: 41.1 ± 3.8 vs. 40.9 ± 4.0 vs. 40.45 ± 13.27 g/L; LDH: 183.4 ± 48.1 vs. 175.5 ± 52.3 vs. 198.76 ± 86.82 U/L; all p > 0.05).

**Figure 2:**
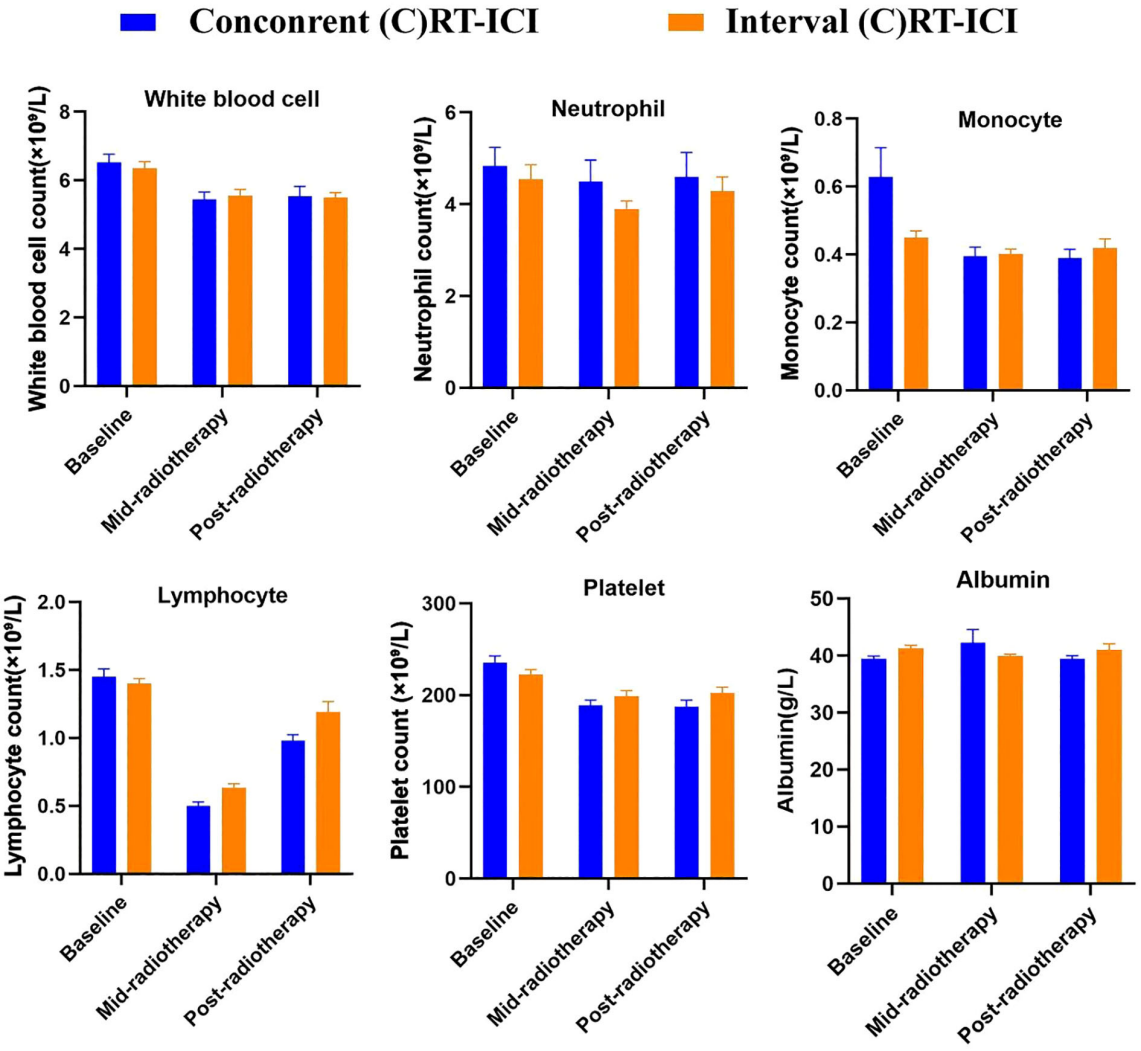
Dynamic hematologic changes during radiotherapy with or without concurrent ICI.

When stratified by the timing of ICI, patients in the concurrent (C)RT-ICI group showed significantly greater reductions in lymphocyte counts at both mid- and post-RT compared with the Interval group. No significant differences were observed in other hematologic parameters (all p > 0.05). Detailed comparisons are provided in **Table 3**, and the dynamic hematologic changes during RT with or without concurrent ICI are illustrated in **Figure 2**.

**Table 3:** Changes in hematologic parameters before, during, and after RT, and the impact of concurrent versus interval radiotherapy and ICI.

Haematological Indexs	Baseline (Mean ± Standard deviation)		P	Mid-RT (Mean ± Standard deviation)		P	Post-RT (Mean ± Standard deviation)		P
	Con (C)RT-ICI	Int (C)RT-ICI		Con (C)RT-ICI	Int (C)RT-ICI		Con (C)RT-ICI	Int (C)RT-ICI	
White blood cell count(×10^9^/L)	6.52 ± 2.87	6.35 ± 2.98	0.576	5.44 ± 2.57	5.55 ± 2.77	0.709	5.54 ± 3.314	5.50 ± 2.12	0.906
Neutrophil count(×10^9^/L)	4.83 ± 4.92	4.54 ± 4.89	0.571	4.49 ± 5.66	3.89 ± 2.56	0.226	4.59 ± 6.281	4.28 ± 4.69	0.587
​Monocyte count(×10^9^/L)	0.62 ± 1.05	0.45 ± 0.31	0.045	0.39 ± 0.33	0.40 ± 0.21	0.802	0.39 ± 0.299	0.42± 0.39	0.448
​Lymphocyte count(×10^9^/L)	1.46 ± 0.709	1.40 ± 0.55	0.423	0.50 ± 0.33	0.63 ± 0.41	0.001	0.98 ± 0.538	1.19 ± 1.14	0.040
Platelet count (×10^9^/L)​	235.65 ± 84.53	222.43 ± 86.82	0.140	189.02 ± 69.14	198.84 ± 89.41	0.259	187.70 ± 80.380	202.40 ± 93.79	0.128
Albumin(g/L)	39.44 ± 5.40	41.27 ± 8.02	0.008	42.25 ± 26.55	39.91 ± 4.31	0.229	39.39 ± 6.517	41.00 ± 15.51	0.262
Lactate dehydrogenase (LDH,U/L)	187.89 ± 72.33	187.87 ± 52.80	0.998	182.47 ± 47.93	190.58 ± 49.73	0.211	197.02 ± 80.310	227.02 ± 264.33	0.279

Con (C)RT-ICI, (Chemo)radiotherapy concurrent immune checkpoint inhibitors.

Int (C)RT-ICI, (Chemo)radiotherapy interval immune checkpoint inhibitors.

### 3.4 Impact of different RT–ICI combinations and hematologic parameters on survival

The median follow-up time for the entire cohort was 34.0 months (95% CI: 29.997–38.003). The median OS was 29.0 months (95% CI: 22.557–35.443), and the median PFS was 18.0 months (95% CI: 14.544–21.456).

**Figure 3:**
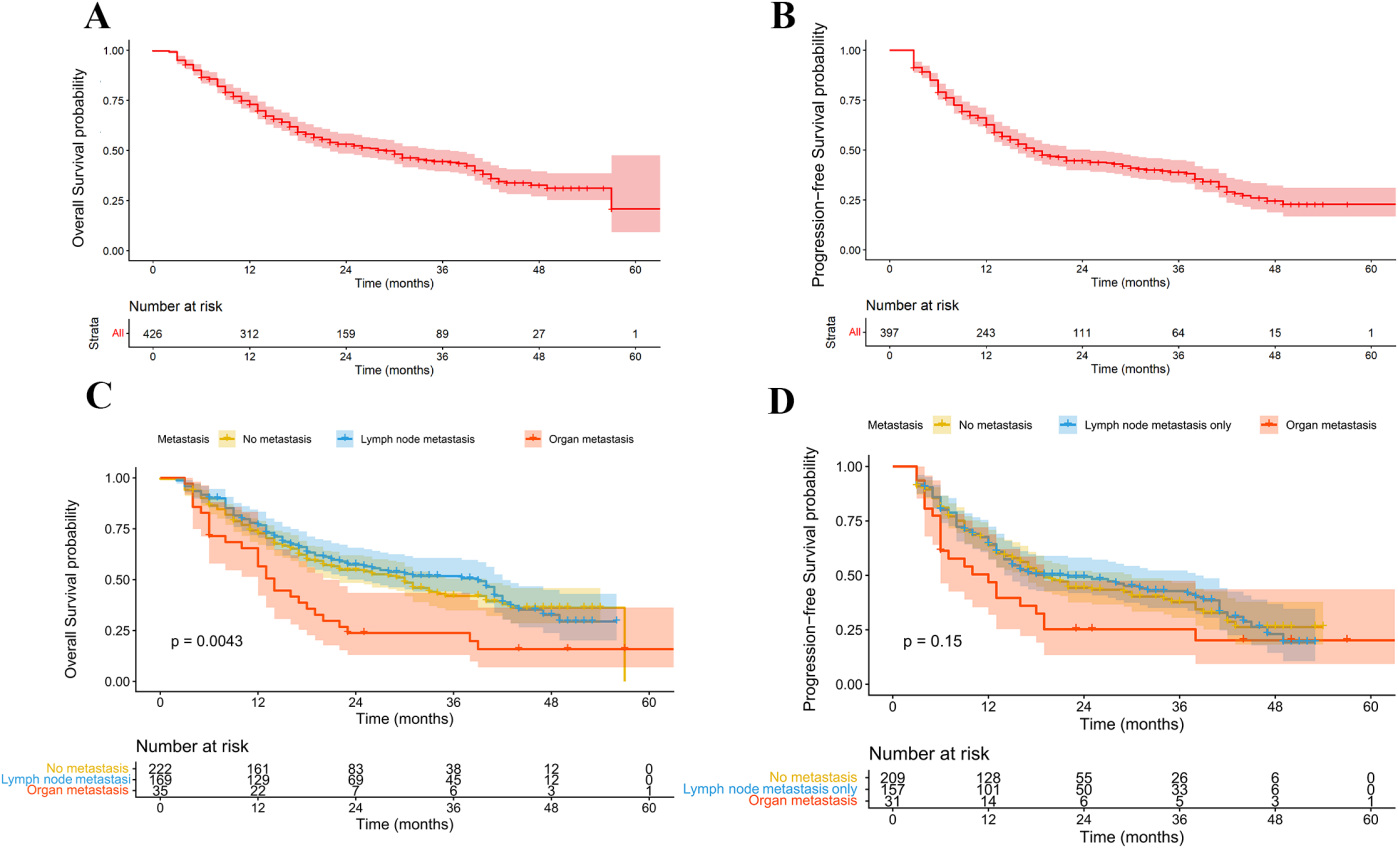
Overall survival (OS) and progression-free survival (PFS) of the entire cohort and the impact of metastatic status. **(A, B)** OS and PFS curves of the whole cohort. **(C, D)** OS and PFS according to metastatic status.

When stratified by metastatic status, patients were categorized into non-metastatic, lymph node-only metastatic, and organ metastatic groups. The median OS was 30.0 months (95% CI: 23.1–36.9) in the non-metastatic group, 39.0 months (95% CI: 29.512–48.531) in the lymph node-only group, and 14.0 months (95% CI: 11.231–16.812) in the organ metastasis group. Patients with organ metastases had significantly worse survival than the other two groups (p = 0.002), while no significant survival difference was observed between the non-metastatic and lymph node-only metastatic groups (p > 0.05). The median PFS was 19.0 months (95% CI: 15.389–22.611) in the non-metastatic group, 22.0 months (95% CI: 11.702–32.298) in the lymph node-only group, and 12.0 months (95% CI: 6.003–17.997) in the organ metastasis group, with no statistically significant differences (**Figure 3**).

**Figure 4:**
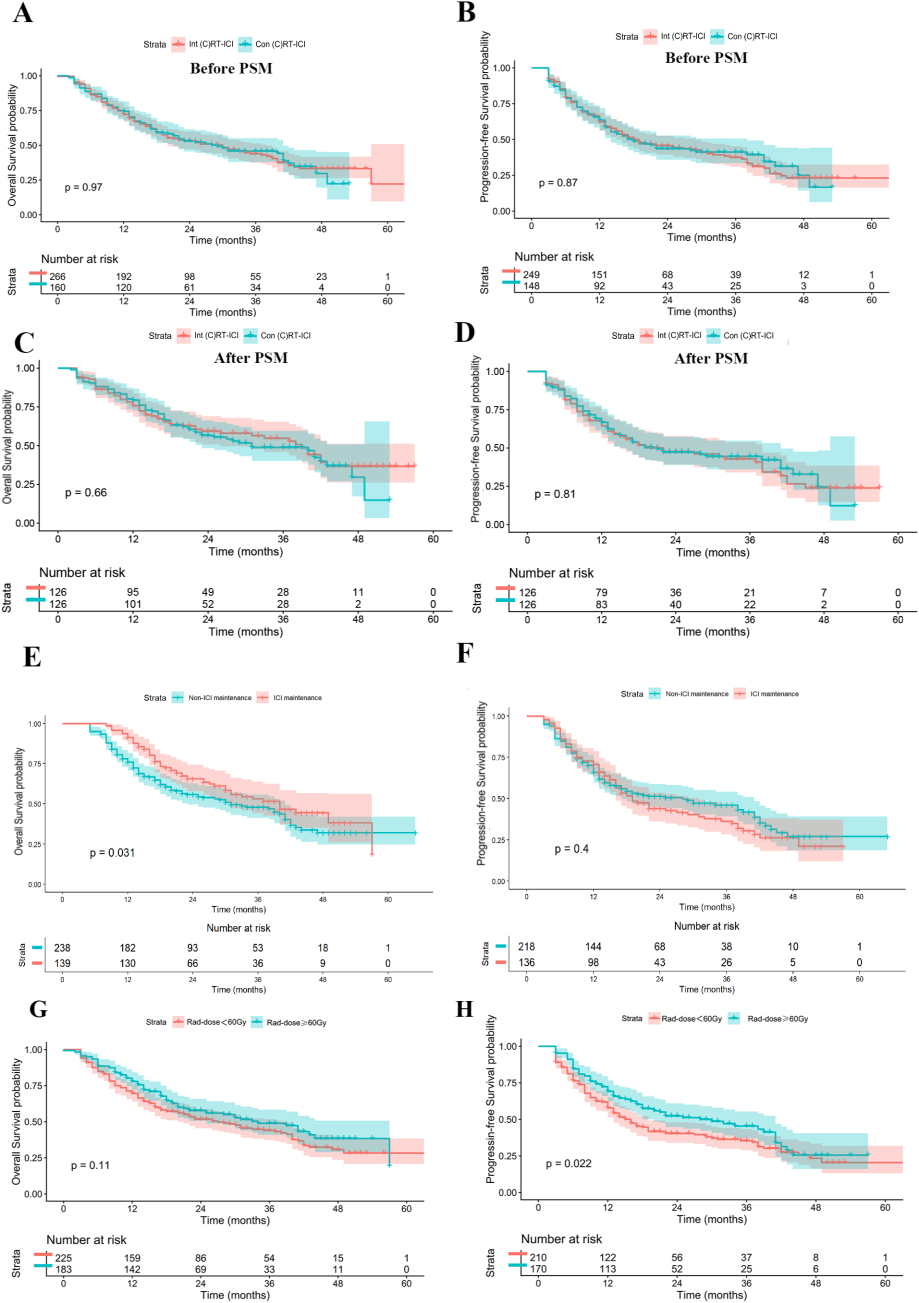
Effects of treatment modalities on overall survival (OS) and progression-free survival (PFS). **(A, B)** Comparison of concurrent (chemo)radiotherapy plus ICI (Con (C)RT-ICI) versus Interval (chemo)radiotherapy plus ICI (Int (C)RT-ICI) before PSM. **(C, D)** Comparison of concurrent (chemo)radiotherapy plus ICI versus Interval (chemo)radiotherapy plus ICI after PSM. **(E, F)** Comparison of the ICI maintenance vs. non-maintenance groups. A landmark analysis at 4 months was employed **(G, H)** Comparison between radiation doses of primary tumor (<60 Gy and ≥60 Gy).

Analysis of different RT-ICI strategies revealed no significant differences in OS or PFS between concurrent and Interval (C)RT-ICI groups (median OS: 30 vs. 29 months; median PFS: 18 vs. 18 months; all p > 0.05), see **Figures 4A, B**. After PSM analysis, the results remained unchanged (**Figures 4C, D**. Baseline characteristics after PSM are shown in **Supplementary Table 1**).

Landmark Analysis was performed for patients who received ICI maintenance, ICI maintenance has better OS compared with those without maintenance (median OS: 40 vs. 31 months, p =0.031), although no PFS advantage was observed (median PFS: 19 vs. 17 months, p = 0.400). For patients receiving RT to the primary esophageal lesion, a radiation dose ≥60 Gy was not associated with superior OS compared with 40–60 Gy (median OS: 34 vs. 27 months, p = 0.113), but it was associated with significantly longer PFS (median PFS: 28 vs. 16 months, p = 0.022). See **Figures 4E–H**.

**Figure 5:**
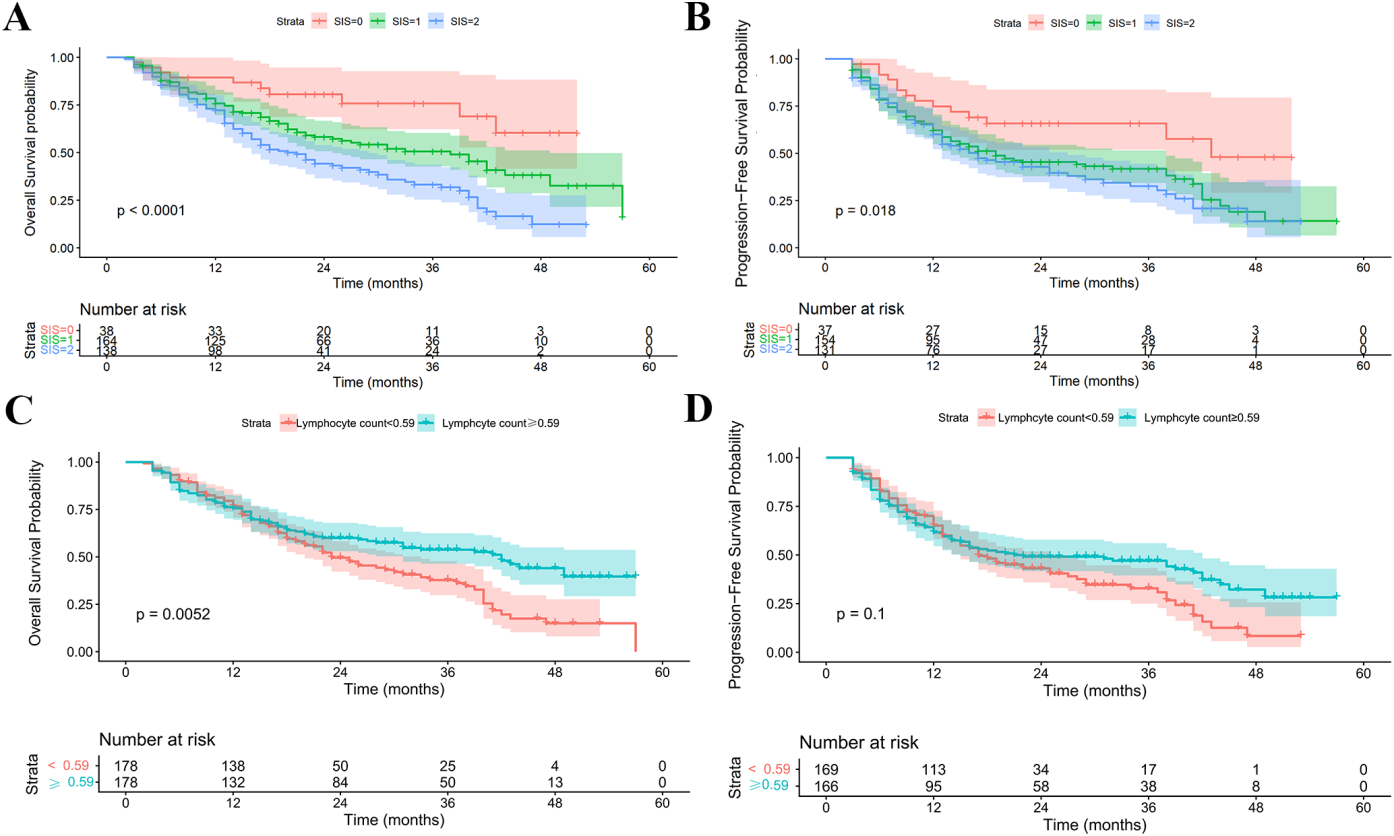
Prognostic impact of hematologic parameters on overall survival (OS) and progression-free survival (PFS). **(A, B)** Mid-radiotherapy lymphocyte counts **(C, D)** Post-radiotherapy SIS values.

Systemic inflammation and nutrition-related indices, including LMR, SIS and PNI, along with other hematologic parameters, were assessed using Cox univariate analysis. The influence of hematologic parameters collected at different timepoints on survival is detailed in **Supplementary Table 2**. Notably, mid-RT lymphocyte counts and post-RT SIS values demonstrated the strongest prognostic associations with OS and PFS. Kaplan-Meier analysis using the median lymphocyte count as the cutoff (0.59 × 10^9^/L) showed significantly longer OS (42 vs. 23 months, p = 0.005) and a trend toward improved PFS (21 vs. 17 months, p = 0.101) in patients with higher counts (>0.59 × 10^9^/L). Post-RT SIS analysis revealed that patients with SIS = 0 had not reached the median OS, whereas those with SIS = 1 and 2 had median OS of 38 and 20 months, respectively (p < 0.001). For PFS, the corresponding median values were 43, 19, and 17 months for SIS = 0, 1, and 2, respectively (p = 0.018). See **Figure 5**.

### 3.5 Univariate and multivariate analyses

To minimize collinearity, only selected hematologic parameters were included in the Cox regression model, specifically baseline SIS, mid-RT lymphocyte counts, and post-RT SIS(**Table 4**).

**Table 4:** Univariate and multivariate analyses of overall survival and progression-free survival.

Demographic factors	OS Univariate analysis		OS Multivariate analysis		PFS Univariate analysis		PFS Multivariate analysis	
	HR (95%CI)	P value	HR (95%CI)	P value	HR (95%CI)	P value	HR (95%CI)	P value
**Gender**		0.017		0.149		0.001	1.660 (1.127-2.445)	0.010
Female	Reference				Reference			
Male	1.471 (1.072-2.018)		1.337 (0.902-1.982)		1.755 (1.264-2.438)			
**Age**		0.188				0.184		
<65	Reference				Reference			
≥65	1.185 (0.920-1.525)				0.838 (0.647-1.087)			
**Smoking**		0.981				0.329		
Yes	Reference				Reference			
No	1.003 (0.772-1.303)				1.141 (0.876-1.487)			
**ECOG score**		0.008		0.991		0.067		0.994
0-1	Reference		Reference		Reference			
≥2	1.340 (1.079-1.665)		0.996 (0.546-1.819)		1.443 (0.975-2.137)		1.002 (0.570-1.761)	
**Tumor Location**		0.278				0.051		0.700
Cervical+Upper	Reference				Reference		1.035 (0.869-1.234)	
Middle+Low	1.087 (0.935-1.263)				1.166 (0.999-1.361)			
**T stage**		0.049		0.689		0.251		
T1-2	Reference		Reference		Reference			
T3-4	1.471 (1.002-2.159)		1.105 (0.677-1.806)		1.232 (0.862-1.761)			
**N stage**		0.244				0.967		
N0	Reference				Reference			
N+	1.285 (0.843-1.959)				0.992 (0.664-1.481)			
**Metastasis status**		0.004		0.002		0.152		0.103
No metastasis	Reference				Reference		Reference	
Lymph node metastasis only	0.920 (0.696-1.215)	0.556	0.906 (0.652-1.259)	0.556	0.978 (0.745-1.284)	0.874	0.867 (0.630-1.194	0.382
Organ metastasis	1.833 (1.208-2.780)	0.004	2.282 (1.369-3.802)	0.002	1.495 (0.954-2.341)	0.079	1.555 (0.912-2.652)	0.105
**Chemotherapy**		0.004		0.026		0.169		
No chemotherapy	Reference				Reference			
Platinum-based doublet	0.451 (0.256-0.792)	0.006	0.322 (0.136-0.764)	0.010	0.502 (0.266-0.950)	0.034		
Monotherapy	0.740 (0.381-1.436)	0.373	0.505 (0.197-1.297)	0.156	0.550 (0.257-1.178)	0.124		
**PD-L1 Status**		0.758						
0	Reference				Reference			
≥1	0.916 (0.523-1.603)				0.971 (0.478-1.971)	0.934		
**ICI drug**		0.713				0.214		
Camrelizumab	Reference				Reference			
Tislelizumab	1.187 (0.770-1.831)	0.438			0.905 (0.565-1.449)	0.677		
Sintilimab	1.001 (0.707-1.417)	0.996			1.233 (0.886-1.714)	0.214		
Pembrolizumab	1.208 (0.768-1.902)	0.413			1.125 (0.693-1.827)	0.634		
Others	1.235 (0.855-1.783)	0.261			1.460 (1.013-2.103)	0.042		
**ICI maintenance**		0.000		0.001		0.934		
No	Reference				Reference			
Yes	0.944 (0.919-0.970)		0.558 (0.394-0.789)		0.989 (0.760-1.287)			
**Modality of Combined RT and ICI**		0.925				0.873		
Concurrent	Reference				Reference			
Interval	0.988 (0.760-1.283)				0.979 (0.751-1.276)			
**Radiation Dose to Primary Esophageal Tumor**		0.118				0.025		0.064
<60 Gy	Reference				Reference		Reference	
≥60 Gy	0.808 (0.619-1.055)				0.735 (0.561-0.963)		0.7450 (0.545-1.018)	
**Baseline SIS**	0.969 (0.945-0.993)	0.011	1.101 (0.858-1.414)	0.449	1.101 (0.858-1.414)	0.534		
**Mid-RT Lymphocyte Count**	0.509 (0.329-0.788)	0.002	0.875 (0.530-1.443)	0.600	0.875 (0.530-1.443)	0.041	0.782 (0.491-1.247)	0.302
**Post-RT SIS**	1.723 (1.359-2.185)	0.000	1.513 (1.154-1.984)	0.003	1.513 (1.154-1.984)	0.013	1.198 (0.939-1.529)	0.147

EOCG, Eastern Cooperative Oncology Group Performance Status; ICI, Immune checkpoint inhibitors, Mid-RT: In the middle of radiotherapy; Post-RT, Post radiotherpay; SIS, Systemic Inflammation Score.

For OS, univariate analysis revealed that male sex, ECOG ≥2, T3–4 stage, presence of visceral metastases, lower mid-RT lymphocyte counts, higher baseline and post-RT SIS values, absence of ICI maintenance, and lack of chemotherapy were significantly associated with worse prognosis (p < 0.05). Multivariate analysis identified ICI maintenance, post-RT SIS, and chemotherapy regimen as independent prognostic factors for OS.

For PFS, univariate analysis indicated that male sex, ECOG ≥2, visceral metastases, lower mid-RT lymphocyte counts, higher post-RT SIS, absence of ICI maintenance, and certain ICI agents were significantly associated with poorer outcomes (p < 0.05). However, in multivariate analysis, only sex remained an independent prognostic factor.

### 3.6 Toxicity.

The analysis of adverse events showed that the overall incidence of grade 1–2 and grade 3–4 toxicities was 41.5% and 36.6%, respectively, among grade 3 adverse events, bone marrow suppression was the most frequent. When stratified by treatment modality, patients receiving concurrent (C)RT-ICI exhibited higher rates of bone marrow suppression, pneumonitis, gastrointestinal reactions, thyroid dysfunction, and esophageal fistula compared with those receiving Interval (C)RT-ICI. However, these differences did not reach statistical significance (all P > 0.05), **Table 5**.

**Table 5:** The toxic effects between Con (C)RT-ICI group and Int (C)RT-ICI group.

Toxic effect	Grade	Con (C)RT-ICI N=160	Int (C)RT-ICI N=266	χ²	P value
Myelosuppression	1-2	30(18.8)	36(13.5)	2.076	0.150
	3-4	71(35.6)	57(26.7)	3.793	0.051
Pneumonitis​	1-2	8(5)	8(3)	1.097	0.295
	3-4	4 (2.5)	4 (1.5)	NA	0.481
Hepatotoxicity	3-4	3 (1.9)	5 (1.9)	NA	1.000
Gastrointestinal toxicity	1-2	45 (28.1)	46 (17.2)	0.633	0.068
	3-4	3 (1.9)	2 (0.8)	NA	0.275
Thyroid dysfunction	1-2	2 (1.3)	2 (0.8)	NA	0.482
Immune-mediated myocarditis	3	0(0)	1 (0.4)	NA	1.000
Immune-mediated hypophysitis	3	0(0)	1 (0.4)	NA	1.000
​Esophageal fistula	/	3(1.9)	2 (0.8)	NA	0.275

Con (C)RT-ICI, (Chemo)radiotherapy concurrent immune checkpoint inhibitors.

Int (C)RT-ICI, (Chemo)radiotherapy interval immune checkpoint inhibitors.

### 3.7 Failure Patterns

A total of 234 patients experienced treatment failure, consisting of 87 in the Con (C)RT-ICI group and 147 in the Int (C)RT-ICI group. Analysis of failure patterns revealed that the local failure rate in the Con (C)RT-ICI group was slightly lower than that in the Int (C)RT-ICI (18.1% vs. 12%), although the difference did not reach statistical significance (**Table 6**).

**Table 6:** Failure patterns.

	Con (C)RT-ICI, N=87 N(%)	Int (C)RT-ICI, N = 147 N(%)	χ²	*P* value
Locoregional Failure	20(12.0)	47(18.1)	2.778	0.096
Distant Metastasis	30(18.7)	34(12.8)	2.267	0.132
Locoregional Failure with Distant Metastasis	37(22.3)	66(25.4)	0.530	0.467

Con (C)RT-ICI, (Chemo)radiotherapy concurrent immune checkpoint inhibitors.

Int (C)RT-ICI, (Chemo)radiotherapy interval immune checkpoint inhibitors.

## 4 Discussion

In this multicenter real-world study, we investigated the prognostic factors and hematologic dynamics in patients with unresectable ESCC treated with RT combined with ICI. We found that concurrent and Interval (C)RT-ICI approaches achieved comparable OS and PFS. However, concurrent (C)RT-ICI was associated with a more pronounced decline in lymphocyte counts and a slightly higher incidence of treatment-related adverse events, though the overall safety profile remained manageable. To our knowledge, this study is the first to provide clinical evidence for oncologists regarding the feasibility of administering RT and ICI concurrently in ESCC. Our results suggest that concurrent (C)RT-ICI may be prioritized for patients with good performance status who wish to expedite the treatment process. Conversely, for patients with poor baseline conditions or comorbidities, withholding ICI during RT or adopting an interval approach appears to be a more prudent strategy.

The optimal integration of RT and ICI in ESCC remains undefined, and related studies are scarce. For example, Duan et al. reported treatment-related adverse events from 86 patients receiving either concurrent or Interval (C)RT-ICI but did not provide comparative survival analyses (10). Evidence from lung cancer indicates that consolidation ICI following CRT, as supported by phase III randomized trials, is currently the standard and should be initiated promptly (11, 12). Nevertheless, smaller studies have suggested that concurrent (C)RT-ICI may slightly increase the risk of adverse events while potentially enhancing therapeutic efficacy (13, 14). Findings from our cohort of 426 patients confirm that concurrent treatment is a relatively safe modality with manageable toxicity, maintaining therapeutic efficacy consistent with the Interval approach.

Importantly, this is the first large-scale study to comprehensively characterize the dynamic changes in hematologic parameters during RT when combined with ICI and to assess their prognostic implications. Previous studies have demonstrated that hematologic indices such as lymphocyte count, SIS, and PNI are associated with prognosis in ESCC patients receiving chemoradiotherapy. However, most of these studies did not account for the dynamic nature of these parameters over the treatment course. In the era of chemoradiotherapy, a decline in lymphocyte counts during RT has been identified as a negative prognostic factor (15, 16), reflecting impaired antitumor immune surveillance. Our study similarly demonstrated that reduced mid-RT lymphocyte counts were associated with poorer prognosis, and that concurrent (C)RT-ICI further exacerbated this decline. Interestingly, in multivariate analysis, mid-RT lymphocyte count was not retained as an independent prognostic factor, a possible explanation is that PD-1/PD-L1 inhibitors can reinvigorate exhausted T-cell populations, thereby mitigating the negative impact of transient lymphopenia on OS (17).

The SIS, which integrates the lymphocyte-to-monocyte ratio and the patient’s nutritional status, has been suggested in previous small-sample studies to correlate with treatment outcomes in ESCC patients receiving chemoradiotherapy and ICI (18). Our findings indicate that, compared with baseline or mid-RT values, the SIS measured 1–2 months after RT was a more stable and independent prognostic factor for both OS and PFS. Since this hematologic parameters are routinely collected during treatment, they are simple, cost-free, and readily applicable in clinical practice, underscoring their considerable translational value.

According to the AJCC 8th edition TNM staging system, distant lymph node metastasis alone is classified as stage IV disease. Although there is currently no universally accepted definition of oligometastatic esophageal cancer, some studies proposal that cases with lymph node–only metastases, regardless of the number of nodes involved, may reasonably be considered to represent an oligometastatic state (19, 20). Interestingly, in our study, patients with isolated lymph node metastasis had a median OS of 39.0 months (95% CI: 29.5–48.5), which was markedly superior to that of patients with visceral metastasis (median 14.0 months, 95% CI: 11.2–16.8), and comparable to patients with locally advanced disease (median OS 30.0 months, 95% CI: 23.1–36.9). Notably, this survival outcome exceeds that reported in most previous studies of oligometastatic ESCC, which generally ranged from 15.9 to 31.3 months (20–24). These results highlight that even among stage IV patients with oligometastatic disease, substantial prognostic heterogeneity persists in the era of combined chemoradiotherapy and ICI.

According to the current NCCN guidelines, the standard treatment for stage IV esophageal cancer is chemotherapy combined with ICI, while the role of RT remains controversial. However, an increasing number of studies have suggested that patients with oligometastatic stage IV disease may benefit from a “triple-modality approach” combining RT, chemotherapy, and ICI (6, 22). Our findings further indicate that patients with isolated distant lymph node metastases appear to derive greater benefit from this triple combination compared with those with other forms of oligometastatic disease. Prospective phase III randomized controlled trials are urgently needed to validate this observation.

It is worth noting that patients receiving ICI maintenance showed significantly improved overall survival. However, this analysis is particularly vulnerable to immortal time bias. To address this, we employed a 4-month landmark analysis, which confirmed that the survival advantage for the maintenance group persisted even after excluding early-event cases. Nevertheless, given the retrospective nature of this multicenter study, potential confounding factors and selection biases remain. Consequently, while our results suggest a positive trend, the survival benefits of ICI maintenance must be interpreted with caution and ultimately require confirmation in prospective randomized controlled trials.

There were some limitations in this study. First, given the retrospective nature of this multicenter study, inherent biases were unavoidable. These include selection bias stemming from heterogeneous treatment regimens and a potential underestimation of adverse events, particularly Grade 1–2 toxicities. Second, since most patients did not undergo testing for PD-L1 expression levels or other immunotherapy-related biomarkers, potential interactions between these biological factors and hematological dynamics could not be evaluated. Third, the majority of enrolled patients had locally advanced disease or limited metastatic disease, restricting the applicability of our findings to patients with extensive multi-organ metastases. Finally, the Interval and Concurrent (C)RT-ICI groups encompassed diverse treatment sequencing patterns, which were not investigated in depth in the current study, future studies with larger cohorts are warranted to more precisely explore optimal sequencing strategies and further enhance the efficacy of combined RT and ICI.

Despite these limitations, the present study has several notable strengths. First, it represents the largest exploratory analysis to date investigating the efficacy and safety of first-line concurrent RT and ICI in patients with ESCC, with a large sample size and comprehensive clinical information that increase the robustness and generalizability of the findings. Second, we systematically collected and analyzed hematological parameters at baseline, during RT, and after RT, generating the largest real-world dataset to date on dynamic hematological changes during RT and ICI. This also allowed us to examine the impact of different RT-ICI patterns on hematological alterations, thereby filling an important knowledge gap. Third, beyond comparing treatment strategies, we systematically assessed the prognostic significance of various common clinicopathological factors and hematological markers. These findings provide important evidence and direction for the design of future phase III prospective clinical trials and contribute to the refinement of comprehensive treatment strategies for esophageal cancer.

## 5 Conclusion

In the setting of combined (C)RT and ICI, ESCC patients with isolated distant lymph node metastasis demonstrated survival outcomes comparable to those without metastasis and significantly better than those with organ metastases. No significant differences in OS or PFS were observed between Concurrent and Interval (C)RT-ICI. Concurrent administration was associated with reduced lymphocyte counts during RT and a slightly higher incidence of adverse events. The SIS value measured 1–2 months after radiotherapy emerged as an important prognostic indicator for ESCC patients receiving (C)RT and ICI.

## Data Availability

The raw data supporting the conclusions of this article will be made available by the authors, without undue reservation.
