# Supplementary material for: Exploratory analysis of prognostic factors and hematologic dynamics in unresectable ESCC treated with concurrent versus interval immune checkpoint inhibitors combined with (chemo)radiotherapy: a multicenter real-world study
Source: Front Immunol. 2026 Mar 12;17:1728912. doi: 10.3389/fimmu.2026.1728912 (PMC13018115; doi:10.3389/fimmu.2026.1728912)
Supplement: Supplementary file 1 [file Table1.docx]

**
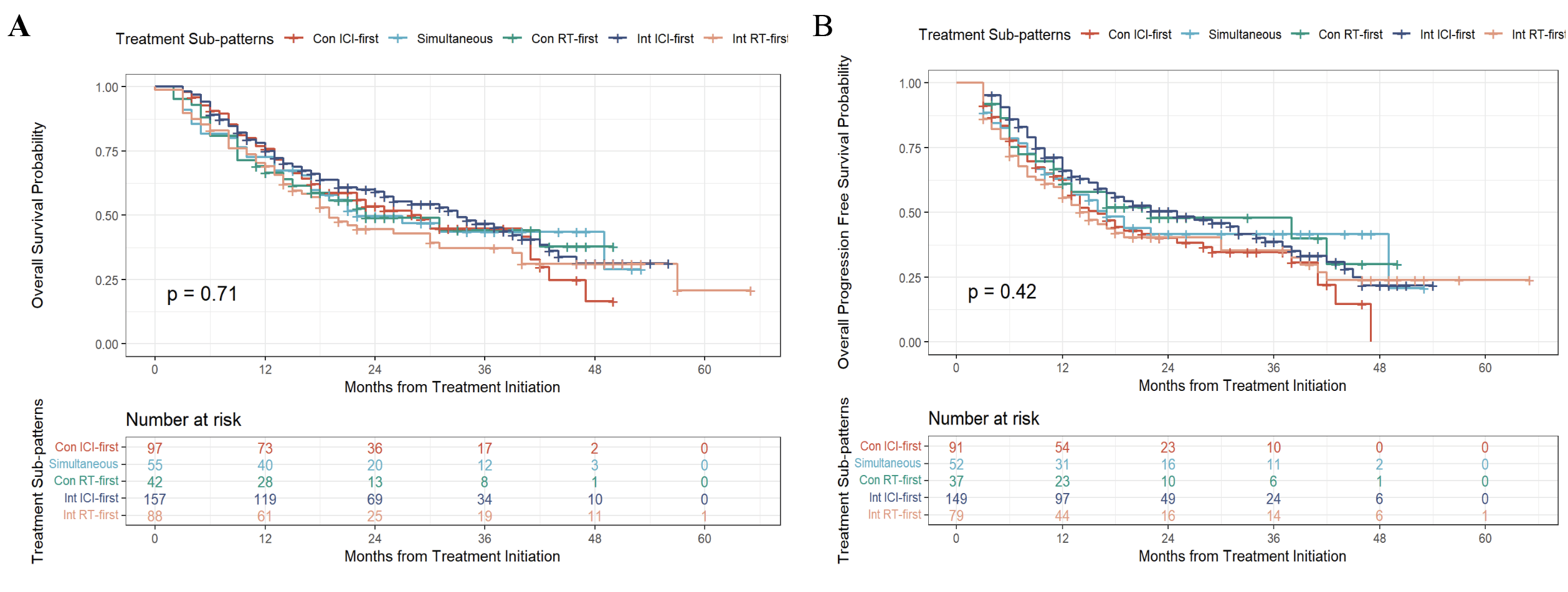
Supplementary Figure S1. Overall Survival(A) and Progression Free Survival (B) Analysis Based on Specific Treatment Sub-patterns.**

Con (C)RT-ICI (Concurrent Group): Includes patients with ICI exposure during the RT course, specifically those starting ICI before RT (ICI-first), simultaneously (Simultaneous), or during the latter part of RT (RT-first).

Int (C)RT-ICI (Interval Group): Includes patients with a distinct treatment-free window between the two modalities, either completing ICI before RT (ICI-first) or starting ICI after RT completion (RT-first).

Supplementary Table 1 Baseline Characteristics after propensity score matching

| **Demographic factors** | Con (C)RT-ICI  N=126 | Int (C)RT-ICI  N=126 | P |
| --- | --- | --- | --- |
| **Gender** |  |  |  |
| Male | 94 (74.60) | 93 (73.81) | 1.00 |
| Female | 32 (25.40) | 33 (26.19) |  |
| **Age** |  |  |  |
| ＜65 | 67 (53.17) | 71 (56.35) | 0.70 |
| ≥65 | 59 (46.83) | 55 (43.65) |  |
| **Smoking** |  |  |  |
| Yes | 65 (51.59) | 77 (61.11) | 0.16 |
| No | 61 (48.41) | 49 (38.89) |  |
| **ECOG score** |  |  |  |
| 0-1 | 115 (91.27) | 118 (93.65) | 0.63 |
| ≥2 | 11 (8.73) | 8 (6.35) |  |
| **Tumor Location** |  |  |  |
| Cervical+Upper | 40 (31.75) | 38 (30.16) | 0.89 |
| Middle+Low | 86 (68.25) | 88 (69.84) |  |
| **T stage** |  |  |  |
| T1-2 | 9 (7.14) | 11 (8.73) | 0.81 |
| T3-4 | 117 (92.86) | 115 (91.27) |  |
| **N stage** |  |  |  |
| N0 | 16 (12.70) | 12 (9.52) | 0.54 |
| N+ | 110 (87.30) | 114 (90.48) |  |
| **Metastasis status** |  |  |  |
| No metastasis | 66 (52.38) | 61 (48.41) | 0.77 |
| Lymph node metastasis only | 52 (41.27) | 55 (43.65) |  |
| Organ metastasis | 8 (6.35) | 10 (7.94) |  |
| **Chemotherapy modality** |  |  |  |
| No chemotherapy | 2 (1.59) | 6 (4.76) | 0.52 |
| Platinum-based doublet | 112 (88.89) | 110 (87.30) |  |
| Others doublet | 1 (0.79) | 1 (0.79) |  |
| Monotherapy | 11 (8.73) | 9 (7.14) |  |
| **ICIs drug** |  |  |  |
| Camrelizumab | 46 (36.51) | 41 (32.54) | 0.31 |
| Tislelizumab | 19 (15.08) | 15 (11.90) |  |
| Sintilimab | 34 (26.98) | 29 (23.02) |  |
| Pembrolizumab | 9 (7.14) | 18 (14.29) |  |
| Others | 18 (14.29) | 23 (18.25) |  |
| **ICIs maintenance** |  |  |  |
| Yes | 50 (39.68) | 53 (42.06) | 0.79 |
| No | 76 (60.32) | 73 (57.94) |  |
| **Radiation Dose to Primary Esophageal Tumor** |  |  |  |
| 40-60 Gy | 75 (59.52) | 72 (57.14) | 0.79 |
| 60 Gy | 51 (40.48) | 54 (42.86) |  |

Con (C)RT-ICI: Chemoradiotherapy concurrent ICIs

Int (C)RT-ICI: Chemoradiotherapy interval ICIs

Supplementary Table 2 COX Univariate analysis for hematologic parameters

| Hematological parameters | OS  Univariate analysis | | PFS  Univariate analysis | |
| --- | --- | --- | --- | --- |
|  | HR (95%CI) | P value | HR (95%CI) | P value |
| **Baseline** |  |  |  |  |
| White blood cell count(×10⁹/L) | 1.028  (0.984-1.074) | 0.217 | 1.002  (0.956-1.049) | 0.948 |
| Neutrophil count(×10⁹/L) | 1.013  (0.989-1.038) | 0.297 | 1.002  (0.973-1.032) | 0.875 |
| ​Monocyte count(×10⁹/L) | 1.066  (0.913-1.244) | 0.418 | 1.025 (0.872-1.204) | 0.768 |
| ​Lymphocyte count(×10⁹/L) | 0.812  (0.646-1.021) | 0.075 | 0.864  (0.691-1.082) | 0.203 |
| Platelet count (×10⁹/L)​ | 1.000  (0.999-1.002) | 0.846 | 1.000  (0.999-1.002) | 0.937 |
| Albumin(g/L) | 0.969  (0.945-0.993) | 0.011 | 1.005  (0.990-1.020) | 0.534 |
| Lactate dehydrogenase (LDH,U/L) | 1.001  (0.999-1.004) | 0.364 | 1.000  (0.998-1.003) | 0.812 |
| LMR | 0.950  (0.902-1.001) | 0.057 | 0.951  (0.903-1.002) | 0.058 |
| SIS | 1.341  (1.097-1.641) | 0.004 | 1.087  (0.896-1.320) | 0.397 |
| PNI | 0.986  (0.973-1.000) | 0.044 | 1.002  (0.989-1.015) | 0.725 |
| **Mid-radiotherapy** |  |  |  |  |
| White blood cell count(×10⁹/L) | 1.052  (1.004-1.102) | 0.034 | 1.015  (0.967-1.066) | 0.551 |
| Neutrophil count(×10⁹/L) | 1.008  (0.982-1.035) | 0.547 | 0.995  (0.963-1.027) | 0.730 |
| ​Monocyte count(×10⁹/L) | 1.235  (0.798-1.910) | 0.344 | 1.196  (0.801-1.785) | 0.382 |
| ​Lymphocyte count(×10⁹/L) | 0.509  (0.329-0.788) | 0.002 | 0.661  (0.445-0.982) | 0.041 |
| Platelet count (×10⁹/L)​ | 1.001  (0.999-1.002) | 0.372 | 1.001  (1.000-1.003) | 0.095 |
| Albumin(g/L) | 1.007  (0.995-1.019) | 0.274 | 0.995  (0.970-1.021) | 0.696 |
| Lactate dehydrogenase (LDH,U/L) | 1.002  (0.999-1.006) | 0.214 | 1.000  (0.996-1.004) | 0.952 |
| LMR | 0.966  (0.915-1.021) | 0.219 | 0.979  (0.932-1.029) | 0.412 |
| SIS | 1.392  (1.047-1.850) | 0.023 | 0.966  (0.741-1260) | 0.797 |
| PNI | 0.995  (0.986-1.003 | 0.211 | 0.993  (0.985-1.001) | 0.092 |
| **Post-radiotherapy** |  |  |  |  |
| White blood cell count(×10⁹/L) | 1.020  (0.983-1.075) | 0.230 | 0.996  (0.948-1.046) | 0.880 |
| Neutrophil count(×10⁹/L) | 0.998  (0.970-1.026) | 0.871 | 0.980(0.944 1.017) | 0.276 |
| ​Monocyte count(×10⁹/L) | 0.990  (0.623-1.571) | 0.965 | 0.909  (0.575-1.436) | 0.681 |
| ​Lymphocyte count(×10⁹/L) | 0.917  (0.755-1.112) | 0.377 | 1.104  (0.934-1.305) | 0.245 |
| Platelet count (×10⁹/L)​ | 1.001  (1.000-1.003) | 0.111 | 1.001  (1.000-1.003) | 0.061 |
| Albumin(g/L) | 0.964  (0.943-0.986) | 0.002 | 0.991  (0.977-1.006) | 0.237 |
| Lactate dehydrogenase (LDH,U/L) | 1.000  (0.999-1.001) | 0.829 | 1.000  (0.998-1.001) | 0.409 |
| LMR | 0.981  (0.954-1.008) | 0.168 | 1.011  (0.994-1.028) | 0.210 |
| SIS | 1.723  (1.359-2.185) | 0.000 | 1.320  (1.059-1.645) | 0.013 |
| PNI | 0.993  (0.985-1.000) | 0.054 | 0.998  (0.991-1.004) | 0.505 |
